# Evaluation of a pre‐exposure prophylaxis programme for men who have sex with men and transgender women in Thailand: learning through the HIV prevention cascade lens

**DOI:** 10.1002/jia2.25540

**Published:** 2020-06-30

**Authors:** Reshmie A Ramautarsing, Ratchadaporn Meksena, Thanthip Sungsing, Tanat Chinbunchorn, Theeranat Sangprasert, Orawan Fungfoosri, Dusita Meekrua, Saman Sumalu, Thapana Pasansai, Witwasin Bunainso, Tashada Wongsri, Nuttakrit Mainoy, Donn Colby, Matthew Avery, Stephen Mills, Ravipa Vannakit, Praphan Phanuphak, Nittaya Phanuphak

**Affiliations:** ^1^ Thai Red Cross AIDS Research Centre PREVENTION Bangkok Thailand; ^2^ Rainbow Sky Association of Thailand Bangkok Thailand; ^3^ Service Workers in Group Foundation Bangkok Thailand; ^4^ Mplus Foundation Chiang Mai Thailand; ^5^ Caremat Foundation Chiang Mai Thailand; ^6^ Thai Red Cross AIDS Research Centre SEARCH Bangkok Thailand; ^7^ FHI 360 Bangkok Thailand; ^8^ Office of Public Health United States Agency for International Development Bangkok Thailand

**Keywords:** HIV prevention, pre‐exposure prophylaxis, Thailand, men who have sex with men, transgender women, prevention cascade

## Abstract

**Introduction:**

Men who have sex with men (MSM) and transgender women (TGW) are two key populations (KPs) in Thailand at high risk for HIV. Uptake and scale‐up of pre‐exposure prophylaxis (PrEP) among them has been slow. We used data from Princess PrEP, Thailand’s largest KP‐led PrEP programme, to operationalize PrEP service cascades. We identified gaps and pointed out where additional data are needed to inform a larger HIV prevention cascade.

**Methods:**

Numbers of people tested for HIV, tested HIV negative, eligible for PrEP (defined as any of the following in the past three months: condomless sex with partners of unknown/uncertain HIV status or antiretroviral treatment or viral load status, multiple partners, engaging in sex work, sexually transmitted infections, injecting drugs, using amphetamine‐type stimulants, or repeated use of post‐exposure prophylaxis), offered PrEP and accepted PrEP during January to November 2019 were retrieved from Princess PrEP database to inform PrEP service cascades for MSM and TGW. Reasons for not accepting PrEP were documented.

**Results:**

Of 6287 MSM who received HIV testing in Princess PrEP, 92.3% were HIV negative and 70.2% of them were eligible for PrEP. PrEP was offered to 94.7% of those eligible and 48.0% of those offered accepted it. Among 900 TGW who had HIV testing, 95.3% tested HIV negative and 64.8% of them met PrEP eligibility criteria. Of these, 95.0% were offered PrEP and 43.9% of them accepted it. Among MSM and TGW who met PrEP eligibility criteria, no or low‐HIV‐risk perception was the most common reason provided (46.7% of 2007 MSM and 41.9% of 296 TGW) for not accepting PrEP.

**Conclusions:**

PrEP service cascades from the Princess PrEP programme identified no or low‐risk perception as key barrier to PrEP acceptance among MSM and TGW who met PrEP eligibility criteria. More implementation research studies are needed to explore PrEP motivation and access in larger communities outside of clinical services. This is to identify gaps and strategies to address them within motivation, access and effective use domains of the HIV prevention cascade.

## 1 INTRODUCTION

Pre‐exposure prophylaxis (PrEP) is extremely effective in reducing HIV acquisition and has resulted in dramatic decreases in new HIV infections when implemented as part of a combination prevention strategy [1, 2]. However, PrEP can only be effective if it is used by those who can benefit from it, and if they use it correctly. For this, demand for PrEP needs to be generated, PrEP needs to be accessible by priority populations, and PrEP use needs to be effective. The HIV prevention cascade, proposed by Schaefer *et al*., is a framework that starts with identifying priority population that could benefit from using an HIV prevention method and consists of these three key domains: motivation for using this HIV prevention method, access to the method and effective use [3]. This cascade allows implementers to identify gaps in biomedical, behavioural and structural aspects of an HIV prevention method, plan interventions to close those gaps and monitor the interventions.

Key population (KPs) at high risk of HIV in Thailand include men who have sex with men (MSM), transgender women (TGW), people who inject drugs (PWID) and female sex workers (FSW). MSM and TGW account for more than half of new HIV infections annually, with HIV prevalence ranging from 17.7% to 28.6% among MSM and 8.8% among TGW in different urban centres [4, 5, 6]. HIV prevention and treatment interventions targeting these KPs are imperative to ending AIDS in Thailand.

As Thailand has included PrEP as part of Universal Health Coverage since October 2019 [7, 8], it has aimed to track the progress and guide the monitoring and evaluation of PrEP scale‐up using the framework of the HIV prevention cascade. Here, we utilized data from the Princess PrEP programme – the largest PrEP programme in Thailand [9] – to operationalize PrEP service cascades for MSM and TGW. We explored gaps identified in these PrEP service cascades, including reasons for not accepting PrEP when offered, among MSM and TGW. We also pointed out where additional data is needed to inform a larger HIV prevention cascade [3]. We hypothesized that there would be gaps identified in PrEP service cascades for MSM and TGW, especially around PrEP uptake and retention. Understanding reasons why MSM and TGW did not accept PrEP when it was offered could guide how PrEP messaging should be reframed. In addition, innovative ways of data collection might be needed to gain more insight about PrEP retention and its effective use.

## 2 METHODS

### 2.1 Programme setting

The Princess PrEP programme is the largest PrEP programme in Thailand. It is part of the Key Populations‐Led Health Services (KPLHS) model, through which trained lay providers, who themselves are members of the KPs they are serving, provide HIV services in community‐based organizations (CBOs) [10]. HIV clinical services under KPLHS included point‐of‐care HIV and sexually transmitted infections (STIs) testing, PrEP and post‐exposure prophylaxis (PEP) dispensing, antiretroviral treatment (ART) service linkages and ART dispensing for stable cases and case management support. A service package was designed by KP communities and co‐delivered by KP lay providers, in close collaboration with public health sectors. For example TGW designed a service package which integrated gender affirming care with sexual health service to ensure that common health concerns prioritized by TGW were addressed.

The KPLHS model, including the Princess PrEP programme, is supported through the President’s Emergency Plan for AIDS Relief (PEPFAR) under the US Agency for International Development (USAID) LINKAGES Project, whereas the PrEP medication itself is provided through the Thai Red Cross Princess Soamsawali HIV Prevention Fund. Through Princess PrEP, KP lay providers have been successfully dispensing free same‐day PrEP since January 2016, contributing to 55% of Thai PrEP users [11]. The programme is currently implemented in six provinces, including Bangkok, Chonburi, Chiang Mai, Chiang Rai, Songkhla and Ubonratchathani, which have high HIV prevalence and incidence among KPs in Thailand [12]. This analysis focused on PrEP services delivered to MSM and TGW between January and November 2019. Routinely collected and de‐identified service data were used. The programme was implemented under a protocol approved by the Institutional Review Board of the Faculty of Medicine, Chulalongkorn University, which waived the need for informed consent from clients to ensure confidentiality and avoid unnecessary disclosure of self‐identity [13].

### 2.2 Data collection for PrEP service cascade

Although the programme started delivering PrEP services to KPs and collecting service data in 2016, data collection to inform PrEP service cascade started in January 2019. Data collection was adjusted at this point to be able to collect number of people tested for HIV, tested HIV negative, eligible for PrEP, offered PrEP and accepted PrEP. Counsellors during a counselling session recorded the data through an electronic case record form. All measures were self‐reported by the client. Routine data quality assurance and improvement was conducted to ensure completeness of data.

Clients who tested HIV negative at any of the CBOs where Princess PrEP was implemented, were assessed for PrEP eligibility defining as any of the following in the previous three months: condomless sex with unknown HIV status partner(s), condomless sex with HIV‐positive partner(s) not on ART or on ART with uncertain viral load status or unknown ART status, having multiple partners, engaging in sex work, symptoms or diagnosis of STIs, injecting drugs, using amphetamine‐type stimulants (ATS), or repeated use of PEP. Eligible clients were offered PrEP by trained counsellors during the post‐test counselling session. PrEP use, safety and efficacy were explained, and clients were asked if they were interested in taking PrEP. Acceptance of PrEP was documented. Those who did not accept PrEP were asked for their reasons by the counsellors. The counsellors then recorded the reasons by selecting them from the list of common reasons in the electronic case record form, which also provided free space to document reasons not included on the list.

Effective use at months 1 and 3 was defined as retention in care at months 1 and 3, as well as self‐reported good adherence during those visits (i.e. at least 4 tablets per week or correct use of on‐demand PrEP for MSM and 7 tablets per week for TGW due to indications that use of feminizing hormone therapy was associated with decreased tenofovir plasma and rectal tissue concentrations) [14, 15].

### 2.3 Statistical analyses

Statistical analyses were performed using Statistics and Data Science (STATA) version 15.1. Demographic data and cascade data were assessed using descriptive statistics as mean, standard deviation, median, interquartile range (IQR) and proportion. T‐test and median test were used for comparison of continuous variables with normal and non‐normal distribution respectively. Chi‐squared test was used to compare categorical variables.

## 3 RESULTS

### 3.1 Men who have sex with men

#### 3.1.1 PrEP service cascade

**Figure 1:**
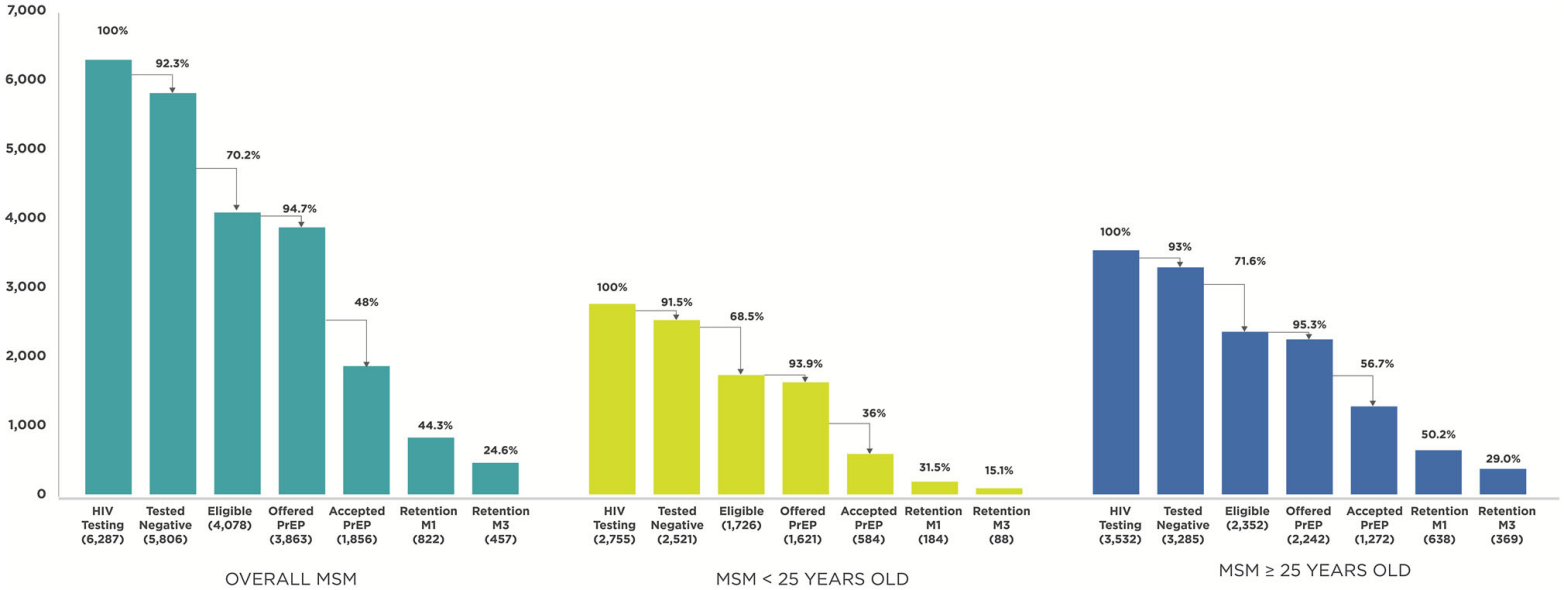
PrEP service cascade for men who have sex with men in the Princess PrEP programme, overall and by age group. PrEP eligibility criteria: Any of the following in the previous three months: condomless sex with unknown HIV status partner(s), condomless sex with HIV‐positive partner(s) not on antiretroviral treatment (ART) or on ART with uncertain viral load status or unknown ART status, having multiple partners, engaging in sex work, symptoms or diagnosis of STIs, injecting drugs, using amphetamine‐type stimulants (ATS), or repeated use of post‐exposure prophylaxis (PEP). PrEP, pre‐exposure prophylaxis; MSM, men who have sex with men; M1, month 1 visit; M3, month 3 visit.

Between 1 January and 30 November 2019, a total of 6287 MSM received HIV testing at one of the sites where Princess PrEP was implemented. Of these, 5806 (92.3%) were HIV negative, of whom 4078 (70.2%) were eligible for PrEP based on the above mentioned criteria. Of these, 3863 (94.7%) were offered PrEP, of whom 1856 (48.0%) accepted PrEP (Figure 1). On‐demand PrEP was chosen at initiation by 4% of MSM. Among those who retained in PrEP service at month 1 (822, 44.3%) and month 3 (457, 24.6%), 99.6% and 99.6% reported effective PrEP use respectively.

When disaggregated by age, MSM younger than 25 years old had lower acceptance of PrEP compared to MSM aged 25 years or older (36.0% vs. 56.7%), and showed lower retention at month 1 (31.5% vs. 50.2%) and month 3 (15.1% vs. 29.0%) (Figure 1).

#### 3.1.2 Focusing on PrEP acceptance

Among 1856 MSM who accepted PrEP, median (IQR) age was 28 (23‐33) years (Table 1). Reported risks in the past three months included having multiple partners in 48.7%, condomless sex with unknown HIV status partner(s) in 48.6%, engaging in sex work in 3.6% and using ATS in 3.5%.

**Table 1:** Demographic and risk characteristics of men who have sex with men and transgender women who were offered PrEP in the Princess PrEP programme from 1 January to 30 November 2019

Demographic and risk characteristics	Characteristics of Men who have sex with men (MSM)					Characteristics of Transgender women (TGW)			
	Offered PrEP (N = 3863)	Did not accept PrEP (N = 2007)	Accepted PrEP (N = 1856)	*p*‐value*	Offered PrEP (N = 528)		Did not accept PrEP (N = 296)	Accepted PrEP (N = 232)	*p*‐value*
Age									
Median (IQR), years	26 (22, 32)	24 (21, 30)	28 (23, 33)	<0.001	25 (21, 30)		24 (20, 29)	26 (23, 30)	0.0180
<25 years old, n (%)	1621 (42%)^a^	1037 (51.7%)^a^	584 (31.6%)^a^	<0.001	248 (47%)		153 (51.7%)	95 (40.9%)	0.0140
Condomless sex with unknown HIV status partner(s), n (%)	1489 (38.5%)	587 (29.2%)	902 (48.6%)	<0.001	200 (37.9%)		107 (36.1%)	93 (40.1%)	0.305
Condomless sex with HIV‐positive partner(s) not on ART or uncertain VL status or unknown ART status, n (%)	73 (1.9%)	10 (0.5%)	63 (3.4%)	<0.001	2 (0.4%)		1 (0.3%)	1 (0.4%)	0.99
Having multiple partners, n (%)	1338 (34.6%)	434 (21.6%)	904 (48.7%)	<0.001	167 (31.6%)		61 (20.6%)	106 (45.7%)	<0.001
Engaging in sex work, n (%)	100 (2.6%)	33 (1.6%)	67 (3.6%)	<0.001	79 (15%)		22 (7.4%)	57 (24.6%)	<0.001
Having STI symptom/diagnosis, n (%)	46 (1.2%)	17 (0.8%)	29 (1.6%)	<0.05	5 (0.9%)		2 (0.7%)	3 (1.3%)	0.658
Injecting substance(s), n (%)	15 (0.4%)	2 (0.1%)	13 (0.7%)	<0.05	0 (0%)		0 (0%)	0 (0%)	–
Using ATS, n (%)	74 (1.9%)	9 (0.4%)	65 (3.5%)	<0.001	3 (0.6%)		2 (0.7%)	1 (0.4%)	0.99
Repeated PEP use, n (%)	29 (0.8%)	2 (0.1%)	27 (1.5%)	<0.001	1 (0.2%)		0 (0%)	1 (0.4%)	0.439

PrEP, pre‐exposure prophylaxis; IQR, interquartile range; THB, Thai Baht (32.5 THB equals 1 US dollar); ART, antiretroviral treatment; VL, viral load; STI, sexually transmitted infection; ATS, amphetamine‐type stimulants; PEP, post‐exposure prophylaxis.

a Missing data for age for 8 MSM who were offered PrEP: 3 who did not accept and 5 who did accept PrEP

b *p*‐values for comparisons made between individuals who did not accept PrEP vs. those who accepted PrEP.

Among 2007 MSM not accepting PrEP, 938 (46.7%) perceived no or low risk, 385 (19.2%) did not want to take pills, 147 (7.3%) wanted to start at a later visit, 142 (7.1%) felt condom use was enough to prevent HIV, 102 (5.1%) could not come back for follow‐up visit, 55 (2.7%) were not interested and 53 (2.6%) were afraid of side effects (Table 2).

**Table 2:** Reasons for not accepting PrEP when offered among men who have sex with men and transgender women in the Princess PrEP programme

Primary reason given for not accepting PrEP	Men who have sex with men (n = 2007)	Transgender women (n = 296)
Perceived no or low risk		
No risk	864 (43.0%)	111 (37.5%)
Low risk	74 (3.7%)	13 (4.4%)
Did not want to take pills	385 (19.2%)	68 (23.0%)
Wanted to start PrEP at a later visit	147 (7.3%)	15 (5.1%)
Felt condom use was enough for HIV prevention	142 (7.1%)	24 (8.1%)
Could not come back for follow‐up visit	102 (5.1%)	17 (5.7%)
Not interested	55 (2.7%)	2 (0.7%)
Afraid of side effects	53 (2.6%)	9 (3.0%)
Others	185 (9.2%)	37 (12.5%)

PrEP, pre‐exposure prophylaxis.

### 3.2 Transgender women

#### 3.2.1 PrEP service cascade

**Figure 2:**
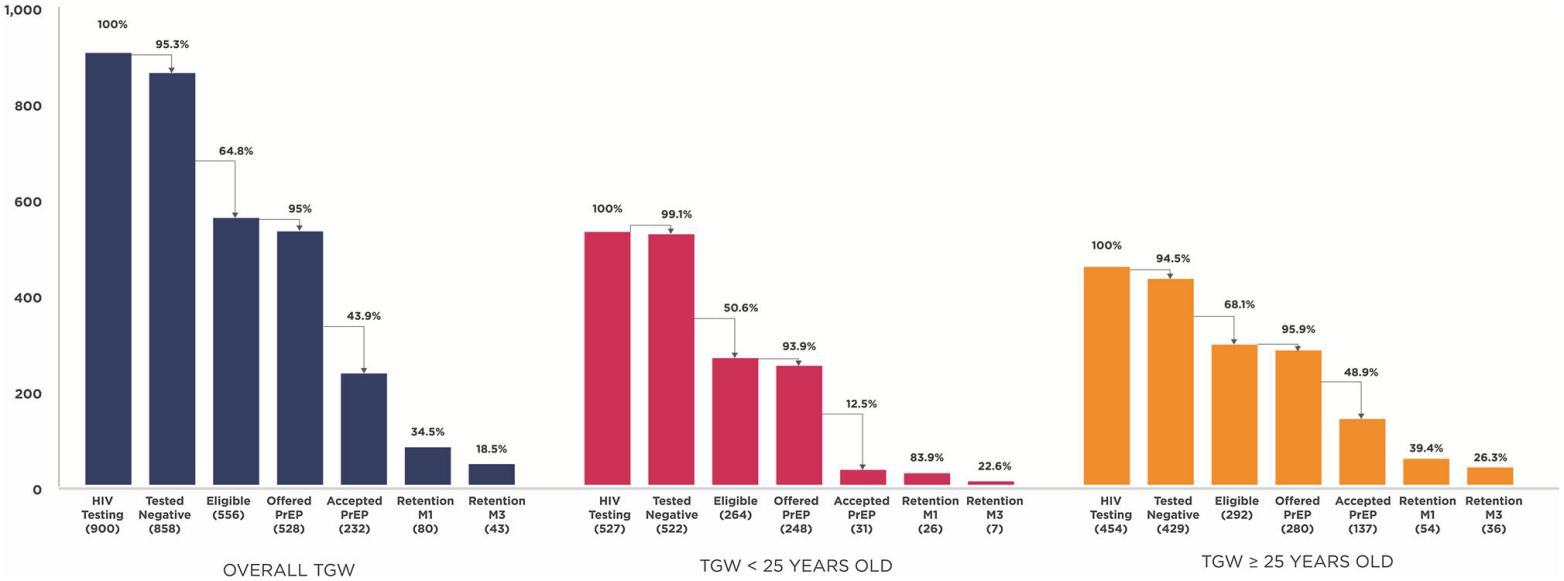
PrEP service cascade for transgender women in Princess PrEP programme, overall and by age group. PrEP eligibility criteria: Any of the following in the previous three months: condomless sex with unknown HIV status partner(s), condomless sex with HIV‐positive partner(s) not on antiretroviral treatment (ART) or on ART with uncertain viral load status or unknown ART status, having multiple partners, engaging in sex work, symptoms or diagnosis of STIs, injecting drugs, using amphetamine‐type stimulants (ATS), or repeated use of post‐exposure prophylaxis (PEP). PrEP, pre‐exposure prophylaxis; TGW, transgender women; M1, month 1 visit; M3, month 3 visit.

A total of 900 TGW received HIV testing between 1 January and 30 November 2019. Of these, 858 (95.3%) were HIV negative, of whom 556 (64.8%) met PrEP eligibility criteria. Of these, 528 (95.0%) were offered PrEP, of whom 232 (43.9%) accepted PrEP (Figure 2). Among those who retained in PrEP service at month 1 (80, 34.5%) and month 3 (43, 18.5%), 98.8% and 100% reported effective PrEP use respectively.

TGW younger than 25 years old had lower acceptance of PrEP compared to those aged 25 years or older (12.5% vs. 48.9%), but showed higher retention at month 1 (83.9% vs. 39.4%) and similar retention at month 3 (22.6% vs. 26.3%) (Figure 2).

#### 3.2.2 Focusing on PrEP acceptance

Of 232 TGW who accepted PrEP, median (IQR) age was 26 (23 – 30) years (Table 1). Reported risks in the past three months included having multiple partners in 45.7%, condomless sex with unknown HIV status partner(s) in 40.1% and engaging in sex work in 24.6%.

Among 296 TGW not accepting PrEP, 124 (41.9%) perceived no or low risk, 68 (23.0%) did not want to take pills, 15 (5.1%) wanted to start at a later visit, 24 (8.1%) felt condom use was enough for HIV prevention, 17 (5.7%) could not come back for follow‐up visit, nine (3.0%) were afraid of side effects and two (0.7%) were not interested (Table 2).

## 4 DISCUSSION

We used year 2019 data from the Princess PrEP programme, which is the largest PrEP programme in Thailand, to demonstrate and explore gaps in PrEP service cascades for MSM and TGW. Around two‐thirds of MSM and TGW in our programme were eligible for PrEP based on risk eligibility criteria recommended in the National Guidelines [16]. An impressive 95% of both MSM and TGW who met PrEP eligibility criteria were offered PrEP, pointing to high level of PrEP service adoption among KP lay providers. However, just less than half of MSM and TGW who were offered PrEP accepted it. Retention in PrEP service was low in both MSM and TGW although almost 100% of those who retained reported effective PrEP use.

We explored further the PrEP acceptance gap and found that almost half of MSM and TGW who met PrEP eligibility criteria perceived themselves as having no or low risk for HIV acquisition. Reframing of messaging around PrEP could significantly impact PrEP uptake. Whereas loss‐framing messages (i.e. emphasizing risks) effectively reaches people who perceive themselves to be at risk, gain‐framing messaging (i.e. emphasizing health) is more effective in reaching those who do not have this risk perception [17]. Focusing PrEP messaging around risk reduction rather than protection, pleasure or sexual health might miss or even distance those populations that would benefit most from PrEP [18]. In addition, we also found that PrEP acceptance among young MSM and TGW was lower than those among their older peers. PrEP messaging may need to be tailored to young KPs as they can often face particular HIV risks due to lower knowledge or lower ability to mitigate those risks [19].

By June 2019, Princess PrEP programme in six provinces accounted for 55% of PrEP users in Thailand, followed by 26% in the PrEP‐15 programme which is a fee‐based, KP‐friendly, PrEP service at the largest HIV testing centre in Bangkok and 19% from the government‐led PrEP service in 25 provinces [11, 20]. PrEP is available for free through the Princess PrEP programme and the government‐led service, using the same eligibility criteria. High PrEP uptake through KP‐led CBOs led the Thai Government to announce the legal endorsement of the roles of KP lay providers in ending AIDS in June 2019. This legal endorsement has allowed HIV clinical services, including PrEP, to be provided by KP lay providers in close collaboration with healthcare providers [21].

We have previously shown that retention in the Princess PrEP programme was lower among TGW compared to MSM, among those younger than 25 years of age, and those with completed education less than bachelor’s degree [9]. Alternative methods of adherence and retention support are needed to tailor interventions to the needs of the clients, including but not limited to mobile health technologies [22]. However, reasons for not retaining in PrEP service should be further explored to assess whether clients not returning for follow‐up visits are still at risk while off PrEP, or if they are simply not at risk anymore. Use of PrEP can be flexible to match periods of different HIV risk, and while PrEP needs to be taken to be effective against HIV acquisition while someone is at risk, it does not need to be used when someone is not at risk [23].

This study has some limitations. Our data were limited to MSM and TGW in six provinces and might not be applicable to other KPs, such as FSW or PWID, or other geographical regions within Thailand. We used self‐report of PrEP adherence which can overestimate the actual use of PrEP. Therefore, effective use of PrEP might be lower than what we reported here [24]. Other measures, including risk behaviours, were also self‐reported which could be biased due to social desirability. We observed that 5% of eligible MSM and TGW were not offered PrEP. However, we did not systematically collect reasons to inform this service gap. In addition, our PrEP service cascades seemed to provide limited data to inform a larger HIV prevention cascade as we did not study PrEP motivation of MSM and TGW who did not access services at community‐based clinics nor the level of access to services among these populations if motivated. According to the HIV prevention cascade, implementers are encouraged to identify gaps in the motivation, access and effective use of an HIV prevention method, plan interventions to close those gaps and monitor the interventions [3]. Ideally, after data on these aspects are applied to the cascade, the proportion of the priority population that is effectively covered by an HIV prevention method can be calculated, which can be used for the monitoring and evaluation of the programme.

Identifying individuals at risk that could benefit from using an HIV prevention method is the first step in operationalizing the HIV prevention cascade. A recent PrEP targets estimation exercise in Thailand revealed an estimated number of Thai people at substantial risk of HIV infection at 148,487 (73,058 – 239,152) in 2020 [25]. Current number of PrEP users in Thailand was reported to be 13,000 – 14,000 or just around 10% of the estimated target [26]. Demand for PrEP, which reflects motivation to use it, is one of the defining factors for the success of PrEP programmes. In the United Kingdom, demand for PrEP has resulted in increased availability of PrEP services, whereas in Australia, demand has secured commitment from policy makers [27, 28]. This level of PrEP demand from potential users has not been evident in Thailand. Future implementation research should therefore be prioritized to study PrEP motivation and access in larger KP communities outside of clinical services.

## 5 CONCLUSIONS

PrEP service cascades from the Princess PrEP programme identified no or low‐risk perception as key barrier to PrEP acceptance among both MSM and TGW who met PrEP eligibility criteria. More implementation research studies are needed to explore PrEP motivation and service access in order to identify gaps and potential strategies to address them within a larger HIV prevention cascade. Definitions of retention and effective use of PrEP need to be further optimized as we gradually understand more the nature of PrEP use among various populations. As Thailand is rolling out and scaling up PrEP as part of its Universal Health Coverage, better data to be applied to the motivation, access and effective use domains of the HIV prevention cascade will allow us to efficiently plan and track progress of PrEP implementation.

## COMPETING INTERESTS

RAR received speakers fees and travel support from Gilead, and speakers fees from Alere. Other authors declare they have no conflicts of interest.

## AUTHORS’ CONTRIBUTIONS

RAR, PP and NP conceptualized the study. RAR, TS, TC, TS(2), OW, PM, SS, TP, WB, TW, DC, MA, SM, RV, PP and NP contributed significantly to the design of the programmes. TS, TC, TS(2), OW, PM, SS, TP, WB and TW contributed significantly to the collection of the data. RM analysed the data. RAR, MA, SM and NP drafted and revised the manuscript. All authors approved the manuscript before submission.
